# Optimization of a Multi–Product Intra-Supply Chain System with Failure in Rework

**DOI:** 10.1371/journal.pone.0167511

**Published:** 2016-12-05

**Authors:** Singa Wang Chiu, Shin-Wei Chen, Chih-Kai Chang, Yuan-Shyi Peter Chiu

**Affiliations:** 1 Dept. of Business Administration, Chaoyang University of Technology, Wufong District, Taichung, Taiwan; 2 Dept. of Industrial Engineering & Management, Chaoyang University of Technology, Wufong District, Taichung, Taiwan; Nankai University, CHINA

## Abstract

Globalization has created tremendous opportunities, but also made business environment highly competitive and turbulent. To gain competitive advantage, management of present-day transnational firms always seeks options to trim down various transaction and coordination costs, especially in the area of controllable intra-supply chain system. This study investigates a multi–product intra-supply chain system with failure in rework. To achieve maximum machine utilization, multiple products are fabricated in succession on a single machine. During the process, production of some defective items is inevitable. Reworking of nonconforming items is used to reduce the quality cost in production and achieving the goal of lower overall production cost. Because reworks are sometimes unsuccessful, failures in rework are also considered in this study. Finished goods for each product are transported to the sales offices when the entire production lot is quality assured after rework. A multi-delivery policy is used, wherein fixed quantity *n* installments of the finished lot are transported at fixed intervals during delivery time. The objective is to jointly determine the common production cycle time and the number of deliveries needed to minimize the long–term expected production–inventory–delivery costs for the problem. With the help of a mathematical model along with optimization technique, the optimal production–shipment policy is obtained. We have used a numerical example to demonstrate applicability of the result of our research.

## Introduction

A multi–product intra-supply chain system with failure in rework is examined in this study. Maximizing machine utilization and minimizing total production and delivery costs are two important operating goals for manufacturing firms today [1–2]. In order to reach the goal of maximum machine utilization, the production planner frequently proposes manufacturing multiple products in succession using single piece of production equipment. Zipkin [3] examined a production system that yields multiple products in large, discrete batches and assumes both the demand and production process to be stochastic. His approach combined standard inventory and queuing sub–models with classical optimization problems to minimize the approximate operating cost of a given facility through the use of certain simple, plausible control policies. Rosenblatt and Rothblum [4] treated capacity as a decision variable when studying multi-item inventory systems under a single–resource capacity constraint. They proposed two solution procedures for deriving an optimal policy within the class of policies that has a fixed cycle for all items with phasing of orders within the cycle. Through illustration of an example, they demonstrated that their solution procedures can be applied to various types of cost functions. Arreola-Risa [5] explored an integrated multi-item production–inventory system with stochastic demands and capacitated production. The objective was to determine the base stock levels needed to minimize the expected inventory costs per unit time. He derived analytical expressions that generate optimal base stock levels for deterministic or exponentially distributed unit manufacturing times. Khoury et al. [6] studied a multi-product lot–scheduling problem characterized by insufficient capacity. A two-product problem was examined using the common cycle approach. Then, they discussed the extended the problem to include any number of products. Caggiano et al. [7] presented a method for computing channel fill rates in a multi-item, multi-echelon service parts distribution system. A simulation technique was used to study the proposed multi-item, three-echelon production–distribution system. Their claimed that their estimation errors were very small over a wide range of base stock level vectors. Björk [8] developed a fuzzy, multi-item economic production quantity (EPQ) model with the aim of helping companies decide production batch sizes under uncertain cycle times. In his model, uncertainty was handled with triangular fuzzy numbers, and an analytical solution to the optimization problem was obtained. Other studies that addressed various aspects of multi-item production planning and optimization issues can be found in [9–14].

For most manufacturing firms today, product quality assurance is an important operational goal. During a given production run, the generation of random defective items is virtually inevitable. Reworking these nonconforming items can serve to increase product quality as well as reduce quality costs in production. Thus, reworking can help minimize overall production and inventory costs, for example, in the production of plastic goods in the plastic injection molding process or in printed circuit board assembly (PCBA) in the PCBA fabrication process. Rework has been adopted by some firms in the manufacturing sector because it increases product quality and decreases costs. Agnihothri and Kenett [15] studied a fabrication system in which all produced items are fully inspected and the identified nonconforming items are reworked. Their objective was to investigate the impact of imperfection on different system performance measures. Subsequently, they provided management guidelines to cope with short- term production control issues (e.g. finding and eliminating bottlenecks) and to achieve the long-term goal of reducing the defective rate. Teunter and Flapper [16] explored a single-stage fabrication system and categorized all fabricated items as perfect quality, re-workable nonconforming, or scrap items. It was assumed that upon production of *N* units, the regular fabrication mode switches to the reworking model and start to repair the nonconforming items. Accordingly, they derived the optimal value for *N* that maximizes the average profit. Sarker et al. [17] studied a multi-stage fabrication system with rework. Two separate reworking policies were examined. The first one assumes that the rework to be done within the same cycle without shortage, and the second policy assumes that the rework to be done after *N* cycles with potential shortages occurrence. They used numerical examples with sensitivity analyses to demonstrate and conclude their research results. Additional studies [18–23] addressed various aspects of imperfect quality production and rework processes.

Although a continuous inventory issuing policy is assumed in the conventional EPQ model [24], multiple or periodic product delivery policies are often used in real–world supply chain environments. Banerjee [25] studied a joint economic lot-size model for purchaser and vendor with a focus on minimizing the joint total relevant cost. He concluded that a joint optimal ordering policy, together with an appropriate price adjustment, could be economically beneficial for both parties. Thomas and Hackman [26] examined a supply chain environment in which a distributor faces price-sensitive demand and has the option of delivery at regular intervals over a finite horizon in exchange for a per-unit cost reduction for units acquired via committed delivery. A simulation approximation is used to develop models for normally distributed demand in order to obtain solutions for the optimal order quantity and a resale price for the distributor. Archetti et al. [27] studied a distribution plan for delivering free newspapers from a production plant to subway, bus, and train stations. Their goals were to minimize the number of vehicle trips needed to distribute all newspapers and the time needed to consume all of the newspapers (i.e., the time needed for readers to receive all of the newspapers). A formulation, several heuristic approaches, and a hybrid method were proposed to solve such an integrated inventory–routing problem with constraints related to the production schedule. Real–world data were applied to their model to demonstrate performance of their approaches. Chiu et al. [28] derived an optimal solution of production cycle length for a multi-product finite production rate system with rework and multi-delivery policy, with the objective of minimizing *vendor*’s total production-inventory costs. Chiu et al. [29] examined an intra-supply chain system wherein *a single product* fabricated by a single machine in production units with prefect rework, and after rework the finished lot is distributed to multiple sales offices under a multi-delivery policy. Other studies [30–49] also addressed different aspects of vendor–buyer integrated types of systems.

With the aim of lowering overall operating cost within an intra-supply chain system [29], this study extends the multi-item finite production rate problem [28] to a multi-product intra-supply chain problem with failures in rework, with the objective is to jointly determine the common production cycle time and number of deliveries needed to minimize the expected production–inventory–delivery costs for the problem. As little attention has been paid to this particular area, our study is intended to link the gap.

## Materials and Methods

### Problem Description

**Fig 1:**
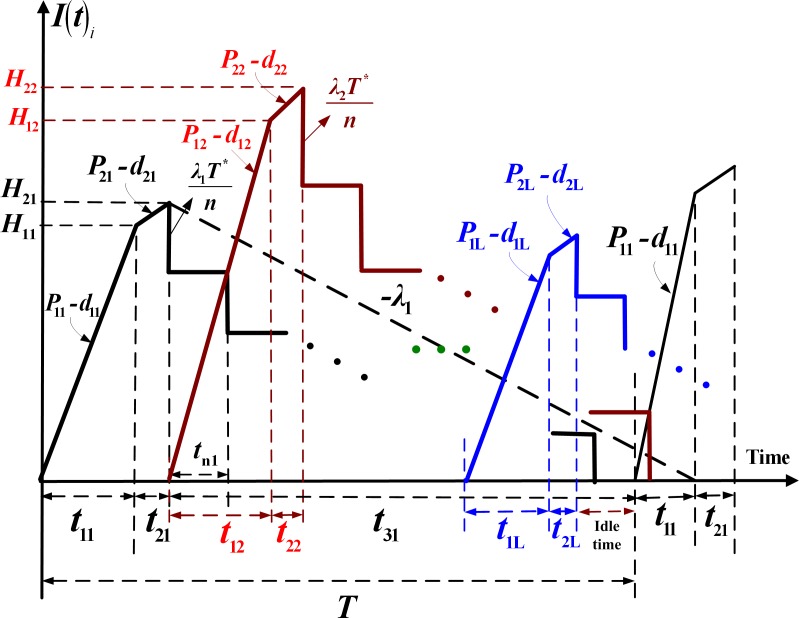
On-hand inventory of perfect quality items in the proposed multi–product intra-supply chain system including stock levels in the production uptime, reworking time, and delivery time

**Fig 2:**
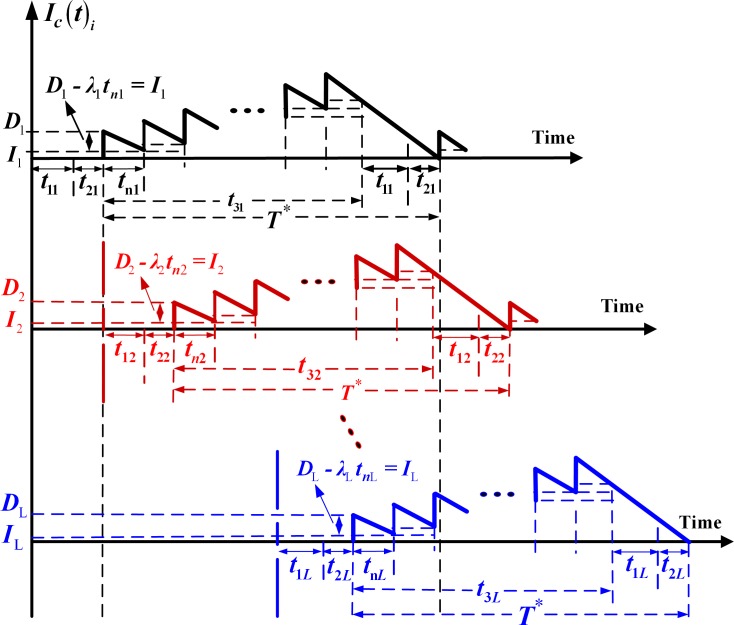
On-hand inventory of product *i* at the sales offices in the proposed multi–product intra-supply chain system including the pile-up stock levels in the end of each delivery

This paper studies a vendor-buyer integrated type of multi–product intra-supply chain system with failure in rework. To achieve the goal of maximizing machine utilization, it is common for the production units to create production plans that involve producing multiple products in sequence on a single machine. In this paper, it is assumed that during the production of each product *i* (where *i* = 1, 2, …, *L*), a portion *x*_i_ of nonconforming items are randomly produced at a rate *d*_1*i*_. All items produced are screened, and inspection cost is included in unit production cost *C*_i_. In operations without permitted shortages, the constant production rate *P*_1i_ of product *i* must satisfy (*P*_1i_ *- d*_1i_ *- λ*_i_) > *0*, where *λ*_i_ is the annual demand rate for product *i*. Therefore, *d*_1i_ can be expressed as *d*_1i_ = *x*_i_*P*_1i_. All nonconforming items are reworked at the rate of *P*_2i_ at the end of the regular production process with additional unit rework cost *C*_Ri_. It is further assumed that a *failure-in-rework* rate *φ*_i_ exists and those that fail during the rework process are scrapped at a disposal cost *C*_Si_ per item. So, the production rate of scrap items during rework *d*_2i_ can be expressed as *φ*_i_*P*_2i_. Finished products *i* are delivered to sales offices only if the entire lot produced is quality assured at the end of the rework process. A discontinuous inventory issuing policy is employed in which a fixed quantity of *n* installments of the finished lot is delivered at fixed time intervals during delivery time *t*_3i_ (see Fig 1). Sales offices’ holding costs (see Fig 2) and product distribution costs are taken into account in the proposed system cost analysis.

**Fig 3:**
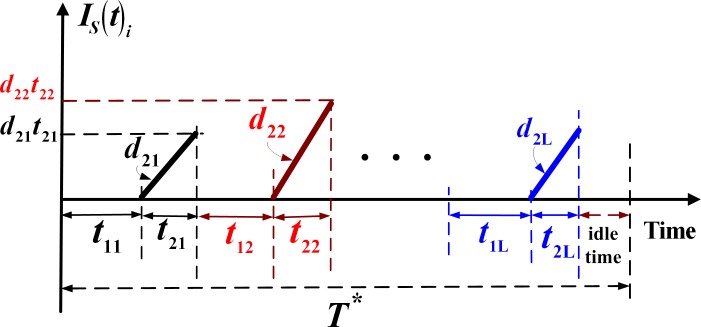
On-hand inventory of scrapped product *i* produced during the rework process in the proposed multi-product intra-supply chain system with failure in rework

To ensure that the production facility has sufficient capacity in regular production and rework processes to satisfy the demands for all *L* products, we must have (see section 3.1 for details): $\sum_{i=1}^{L}\left\{\left[\lambda_{i}/\left(1-\varphi_{i}x_{i}\right)\right]/P_{1i}+x_{i}\left[\lambda_{i}/\left(1-\varphi_{i}x_{i}\right)\right]/P_{2i}\right\}<1$. The level of on-hand inventory of scrapped product *i* produced during the rework process is illustrated in Fig 3.

The cost-related parameters for each product *i* include production setup cost *K*_i_, unit stock holding cost *h*_i_, holding cost *h*_1i_ for each item in rework, fixed distribution cost *K*_1*i*_ per shipment, unit shipping cost *C*_Ti_, and sales offices’ unit stock holding cost *h*_2*i*_. Additional notation also includes in the Appendix A.

### Mathematical Modeling

By examining Figs 1–3, one can directly obtain the following formulae:

$$t_{1i}=\frac{Q_{i}}{P_{1i}}=\frac{H_{1i}}{P_{1i}-d_{1i}}$$ (1)

$$t_{2i}=\frac{x_{i}Q_{i}}{P_{2i}}$$ (2)

$$t_{3i}=nt_{ni}=T-\left(t_{1i}+t_{2i}\right)$$ (3)

$$T=t_{1i}+t_{2i}+t_{3i}$$ (4)

$$d_{1i}t_{1i}=x_{i}Q_{i}$$ (5)

$$H_{1i}=\left(P_{1i}-d_{1i}\right)t_{1i}$$ (6)

$$H_{2i}=H_{1i}+\left(P_{2i}-d_{2i}\right)t_{2i}$$ (7)

It is noted that in Eq (1), *Q*_*i*_ denotes the production batch size per cycle for product *i*, *P*_1*i*_ represents the constant production rate for product *i*, and hence *t*_1*i*_ is the production uptime needed to produce the batch size *Q*_*i*_ of product *i* in a cycle. In Eq (2), *x*_*i*_*Q*_*i*_ means the total number of defective items of product *i* produced in a production cycle, *P*_2*i*_ represents reworking rate of product *i*, and hence *t*_2*i*_ is the reworking time needed to repair these defective items of product *i* in a cycle. Eq (3) indicates that during the delivery time *t*_3*i*_, all perfect quality items of product *i* start to be distributed to sales office in *n* fixed-quantity installments of the batch size, at fixed intervals *t*_n*i*_ of delivery time. Eq (5) shows that the relationship between the production rate *d*_1*i*_ of defective items of product *i* in uptime and the total number of defective items produced for product *i*. Eq (6) represents the on-hand inventory level of product *i* when regular production ends and how it can be computed. Eq (7) indicates the maximum on-hand inventory level of product *i* when reworking process finishes and how it can be calculated.

The holding cost for finished product *i* during *t*_3_, where *n* fixed-quantity installments of the finished batch are distributed at a fixed interval of time [32] is

$$h_{i}\left(\frac{n-1}{2n}\right)H_{2i}t_{3i}$$ (8)

Total *n* shipment delivery cost for product *i* in a cycle is

$$nK_{1i}+C_{Ti}Q_{i}\left(1-\varphi_{i}x_{i}\right)$$ (9)

From Fig 2, because *n* installments (fixed quantity *D*) of the finished lot are delivered to a sales office at a fixed interval of time *t*_n*i*_, one has the following:

$$D_{i}=\frac{H_{2i}}{n}$$ (10)

$$t_{ni}=\frac{t_{3i}}{n}$$ (11)

$$I_{i}=D_{i}-\lambda_{i}t_{ni}$$ (12)

The holding cost for finished product *i* stored at the sales offices, is [12]

$$h_{2i}\left[n\frac{\left(D_{i}-I_{i}\right)}{2}t_{ni}+\frac{nI_{i}}{2}(t_{1i}+t_{2i})+\frac{n(n+1)}{2}I_{i}t_{ni}\right]$$ (13)

Eq (8) shows the total holding cost for finished product *i* during *t*_3_ [32]. Eq (9) gives the total delivery cost per cycle which includes the fixed and the variable transportation costs for all products. Eq (10) shows the fixed quantity per shipment of product *i* and how it can be computed and Eq (11) gives how to obtain the fixed interval of time *t*_*ni*_ in delivery time *t*_3*i*_ for product *i*. Eq (12) represents the number of items of product *i* left (after satisfying the demand) in the end of each delivery at the sale offices. Eq (13) shows how to calculate the stock holding costs at the sales offices [12].

Total production-inventory-delivery cost per cycle *TC*(*Q*_i_, *n*) for *i* = 1, 2, …, *L*, consists of the production setup cost, variable production cost, variable reworking and disposal costs, fixed and variable delivery costs, production units’ holding cost of perfect quality items during *t*_1*i*_, *t*_2*i*_, and *t*_3*i*_, holding cost of nonconforming items in *t*_1*i*_, holding cost of reworked items at *t*_2*i*_, and sales offices’ holding cost of product *i*. Therefore, total *TC*(*Q*_i_, *n*) for *L* products is

$$\sum_{i=1}^{L}TC\left(Q_{i},n\right)=\sum_{i=1}^{L}\left\{\begin{array}{l} K_{i}+C_{i}Q_{i}+C_{Ri}\left(x_{i}Q_{i}\right)+C_{Si}\varphi_{i}\left(x_{i}Q_{i}\right)+nK_{1i}+C_{Ti}\left[Q_{i}\left(1-\varphi_{i}x_{i}\right)\right]\\ +h_{i}\left[\frac{H_{1i}+d_{1i}t_{1i}}{2}\left(t_{1i}\right)+\frac{H_{1i}+H_{2i}}{2}\left(t_{2i}\right)+\frac{n-1}{2n}(H_{2i}t_{3i})\right]\\ +h_{1i}\frac{d_{1i}t_{1i}}{2}\left(t_{2i}\right)+h_{2i}\left[n\frac{\left(D_{i}-I_{i}\right)}{2}t_{ni}+\frac{nI_{i}}{2}(t_{1i}+t_{2i})+\frac{n(n+1)}{2}I_{i}t_{ni}\right] \end{array}\right\}$$ (14)

By taking the randomness of defective rate *x* into account, our cost analysis uses the expected values of *x*. Substituting all variables from Eqs (1) to (13) in Eq (14) and applying the renewal reward theorem and with further derivations, E[*TCU*(*Q*_i_,*n*)] can be obtained as follows:

$$E\left[TCU\left(Q_{i},n\right)\right]=\sum_{i=1}^{L}\frac{1}{\left(1-\varphi_{i}E[x_{i}]\right)}\left\{\begin{array}{l} \left[C_{i}\lambda_{i}+\frac{K_{i}\lambda_{i}}{Q_{i}}+C_{Ri}\lambda_{i}E[x_{i}]+C_{Si}\varphi_{i}E[x_{i}]\lambda_{i}+C_{Ti}\lambda_{i}+\frac{nK_{1i}\lambda_{i}}{Q_{i}}\right]\\ +\frac{h_{i}Q_{i}\lambda_{i}}{2}\left[\begin{array}{l} \left(\frac{1}{\lambda_{i}}-\frac{1}{\lambda_{i}n}+\frac{1}{P_{1i}n}\right)+E[x_{i}]\left(\frac{1}{P_{2i}}+\frac{1}{P_{2i}n}\right)-E{\left[x_{i}\right]}^{2}\left(\frac{1}{P_{2i}}+\frac{\varphi_{i}}{P_{2i}n}\right)\\ +\left(1-\frac{1}{n}\right)\left[E[x_{i}]\left(\frac{\varphi_{i}}{P_{1i}}-\frac{2\varphi_{i}}{\lambda_{i}}\right)+E{\left[x_{i}\right]}^{2}\left(\frac{\varphi_{i}^{2}}{\lambda_{i}}\right)\right] \end{array}\right]\\ +\frac{h_{1i}Q_{i}\lambda_{i}E{\left[x_{i}\right]}^{2}}{2P_{2i}}+\frac{h_{2i}Q_{i}\lambda_{i}(1-\varphi_{i}E[x_{i}])}{2}\left[\frac{\left(1-\varphi_{i}E[x_{i}]\right)}{\lambda_{i}n}+\left(1-\frac{1}{n}\right)\left[\frac{1}{P_{1i}}+\frac{E[x_{i}]}{P_{2i}}\right]\right] \end{array}\right\}$$ (15)

As $Q_{i}=\frac{T\lambda_{i}}{1-\varphi_{i}E[x_{i}]}$, and let $E_{0i}=\frac{1}{1-\varphi_{i}E\left[x_{i}\right]}$ and $E_{1i}=\frac{E\left[x_{i}\right]}{1-\varphi_{i}E\left[x_{i}\right]}$, from Eq (15) one obtains the total expected system cost per unit time for producing *L* products, E[*TCU*(*T*, *n*)] as

$$E\left[TCU\left(T,n\right)\right]=\sum_{i=1}^{L}\left\{\begin{array}{l} \left[C_{i}\lambda_{i}E_{0i}+\frac{K_{i}}{T}+C_{Ri}\lambda_{i}E_{1i}+C_{Si}\varphi_{i}\lambda_{i}E_{1i}+C_{Ti}\lambda_{i}+\frac{nK_{1i}}{T}\right]\\ +\frac{h_{i}\lambda_{i}^{2}}{2}T\left[\frac{1}{\lambda_{i}}+\frac{\varphi_{i}E_{0i}E_{1i}}{P_{1i}}+\frac{E_{0i}E_{1i}-E_{1i}{}^{2}}{P_{2i}}\right]+\frac{h_{1i}\lambda_{i}^{2}E_{1i}^{2}}{2P_{2i}}T\\ +\frac{h_{2i}\lambda_{i}^{2}}{2}T\left(\frac{E_{0i}}{P_{1i}}+\frac{E_{1i}}{P_{2i}}\right)+\frac{\lambda_{i}^{2}}{2n}T\left[\frac{1}{\lambda_{i}}-\frac{E_{0i}}{P_{1i}}-\frac{E_{1i}}{P_{2i}}\right]\left(h_{2i}-h_{i}\right) \end{array}\right\}$$ (16)

## Results and Discussion

### Joint Determination of Cycle Time and Shipment Policy

To determine the optimal rotation cycle time T* and number of shipments n*, one first proves that the expected system cost E[*TCU*(*T*, *n*)] is convex. Applying the Hessian matrix equations [50] and ensuring that the following condition holds:

$$\left[\begin{array}{cc} T & n \end{array}\right]\cdot \left(\begin{array}{cc} \frac{\partial^{2}E\left[TCU\left(T,n\right)\right]}{\partial T^{2}} & \frac{\partial^{2}E\left[TCU\left(T,n\right)\right]}{\partial T\partial n}\\ \frac{\partial^{2}E\left[TCU\left(T,n\right)\right]}{\partial T\partial n} & \frac{\partial^{2}E\left[TCU\left(T,n\right)\right]}{\partial n^{2}} \end{array}\right)\cdot \left[\begin{array}{c} T\\ n \end{array}\right]>0$$ (17)

From Eq (16) one has:

$$\frac{\partial E\left[TCU(T,n)\right]}{\partial T}=\sum_{i=1}^{L}\left\{\begin{array}{l} -\frac{K_{i}}{T^{2}}-\frac{nK_{1i}}{T^{2}}+\frac{h_{i}\lambda_{i}^{2}}{2}\left[\frac{1}{\lambda_{i}}+\frac{\varphi_{i}E_{0i}E_{1i}}{P_{1i}}+\frac{E_{0i}E_{1i}-E_{1i}{}^{2}}{P_{2i}}\right]+\frac{h_{1i}\lambda_{i}^{2}E_{1i}^{2}}{2P_{2i}}\\ +\frac{h_{2i}\lambda_{i}^{2}}{2}\left(\frac{E_{0i}}{P_{1i}}+\frac{E_{1i}}{P_{2i}}\right)+\frac{\lambda_{i}^{2}}{2n}\left[\frac{1}{\lambda_{i}}-\frac{E_{0i}}{P_{1i}}-\frac{E_{1i}}{P_{2i}}\right]\left(h_{2i}-h_{i}\right) \end{array}\right\}$$ (18)

$$\frac{\partial^{2}E\left[TCU\left(T,n\right)\right]}{\partial T^{2}}=\sum_{i=1}^{L}\left[\frac{2\left(K_{i}+nK_{1i}\right)}{T^{3}}\right]$$ (19)

$$\frac{\partial E\left[TCU\left(T,n\right)\right]}{\partial n}=\sum_{i=1}^{L}\left[\frac{K_{1i}}{T}+\frac{\lambda_{i}^{2}}{2n^{2}}T\left(\frac{1}{\lambda_{i}}-\frac{E_{0i}}{P_{1i}}-\frac{E_{1i}}{P_{2i}}\right)\left(h_{i}-h_{2i}\right)\right]$$ (20)

$$\frac{\partial^{2}E\left[TCU\left(T,n\right)\right]}{\partial n^{2}}=\sum_{i=1}^{L}\left[-\frac{\lambda_{i}^{2}T}{n^{3}}\left(\frac{1}{\lambda_{i}}-\frac{E_{0i}}{P_{1i}}-\frac{E_{1i}}{P_{2i}}\right)\left(h_{i}-h_{2i}\right)\right]$$ (21)

$$\frac{\partial E\left[TCU\left(T,n\right)\right]}{\partial T\partial n}=\sum_{i=1}^{L}\left[-\frac{K_{1i}}{T^{2}}+\frac{\lambda_{i}^{2}}{2n^{2}}\left(\frac{1}{\lambda_{i}}-\frac{E_{0i}}{P_{1i}}-\frac{E_{1i}}{P_{2i}}\right)\left(h_{i}-h_{2i}\right)\right]$$ (22)

Substituting Eqs (19), (21) and (22) in Eq (17) and with further derivation gives

$$\left[\begin{array}{cc} T & n \end{array}\right]\cdot \left(\begin{array}{cc} \frac{\partial^{2}E\left[TCU\left(T,n\right)\right]}{\partial T^{2}} & \frac{\partial^{2}E\left[TCU\left(T,n\right)\right]}{\partial T\partial n}\\ \frac{\partial^{2}E\left[TCU\left(T,n\right)\right]}{\partial T\partial n} & \frac{\partial^{2}E\left[TCU\left(T,n\right)\right]}{\partial n^{2}} \end{array}\right)\cdot \left[\begin{array}{c} T\\ n \end{array}\right]=\sum_{i=1}^{L}\frac{2K_{i}}{T}>0$$ (23)

Eq (23) yields positive results, because *K*_*i*_ and *T* are both positive. It follows that E[*TCU*(*T*, *n*)] is a strictly convex function for all *T* and *n* values other than zero. Therefore, there exists a minimum for E[*TCU*(*T*, *n*)].

Then, to jointly determine rotation cycle time T* and number of shipments n*, one differentiates *E[TCU(T*, *n)]* with respect to *T* and *n*, and solve the linear systems of Eqs (18) and (20) by setting these partial derivatives equal to zero. With further derivations, one obtains:

$$T*=\sqrt{\frac{2\sum_{i=1}^{L}\left(K_{i}+nK_{1i}\right)}{\sum_{i=1}^{L}\left\{\begin{array}{l} h_{i}\lambda_{i}^{2}\left[\frac{1}{\lambda_{i}}+\frac{\varphi_{i}E_{0i}E_{1i}}{P_{1i}}+\frac{E_{0i}E_{1i}-E_{1i}{}^{2}}{P_{2i}}\right]+\frac{h_{1i}\lambda_{i}^{2}E_{1i}^{2}}{P_{2i}}\\ +h_{2i}\lambda_{i}^{2}\left(\frac{E_{0i}}{P_{1i}}+\frac{E_{1i}}{P_{2i}}\right)+\frac{\lambda_{i}^{2}}{n}\left[\frac{1}{\lambda_{i}}-\frac{E_{0i}}{P_{1i}}-\frac{E_{1i}}{P_{2i}}\right]\left(h_{2i}-h_{i}\right) \end{array}\right\}}}$$ (24)

and

$$n*=\sqrt{\frac{\sum_{i=1}^{L}K_{i}\cdot \sum_{i=1}^{L}\left[\lambda_{i}^{2}\left(h_{2i}-h_{i}\right)\left(\frac{1}{\lambda_{i}}-\frac{E_{0i}}{P_{1i}}-\frac{E_{1i}}{P_{2i}}\right)\right]}{\left(\sum_{i=1}^{L}K_{1i}\right)\cdot \sum_{i=1}^{L}\left[h_{i}\lambda_{i}^{2}\left(\frac{1}{\lambda_{i}}+\frac{E_{0i}E_{1i}-E_{1i}{}^{2}}{P_{2i}}+\frac{\varphi_{i}E_{0i}E_{1i}}{P_{1i}}\right)+\frac{h_{1i}\lambda_{i}^{2}E_{1i}{}^{2}}{P_{2i}}+h_{2i}\lambda_{i}^{2}\left(\frac{E_{0i}}{P_{1i}}+\frac{E_{1i}}{P_{2i}}\right)\right]}}$$ (25)

### Prerequisite Condition

One prerequisite condition must be satisfied (i.e., Eq (26)) to ensure that the machine in the proposed multi-product manufacturing system has sufficient capacity to manufacture and rework *L* different products under the rotation cycle time policy.

$$\sum_{i=1}^{L}\left[\left(\frac{\lambda_{i}E_{0i}}{P_{1i}}\right)+\left(\frac{\lambda_{i}E_{1i}}{P_{2i}}\right)\right]<1$$ (26)

The setup time for each product will be another factor to carefully consider. In general, production setup time is relatively short compared to the total production and rework times. However, if the setup time becomes a factor, there must be enough time in each cycle to account for the sum of the production, rework, and setup times to produce *L* products [2]. Let *S*_*i*_ denote the setup time for product *i*, then the following condition must hold:

$$\sum_{i=1}^{L}\left[S_{i}+\left(\frac{Q_{i}}{P_{1i}}\right)+\left(\frac{Q_{i}E[x_{i}]}{P_{2i}}\right)\right]<T$$ (27)

As *Q*_*i*_ = *Tλ*_*i*_*E*_0*i*_, Eq (27) can be rearranged as

$$T>\frac{\sum_{i=1}^{L}S_{i}}{1-\sum_{i=1}^{L}\left[\lambda_{i}\left(\frac{E_{0i}^{}}{P_{1i}}+\frac{E_{1i}}{P_{2i}}\right)\right]}=T_{\min}$$ (28)

Therefore, to include setup times in the proposed model, one should choose the optimal rotation cycle time from max(T*, *T*_min_) [2].

### Numerical Example

Suppose in a multi-product intra-supply chain system, there are five different products to be made on a single machine under a rotation cycle time policy. Annual demands *λ*_*i*_ for these products are 3000, 3200, 3400, 3600, and 3800, respectively. They can be manufactured at annual production rates *P*_1*i*_ 58000, 59000, 60000, 61000, and 62000, respectively. All items produced are screened and the inspection cost is included in unit production cost. During production, there are random defective rates associated with these products and they follow a uniform distribution over intervals of [0, 0.05], [0, 0.10], [0, 0.15], [0, 0.20], and [0, 0.25]. All defective items are reworked at an annual rate *P*_2i_ at the end of production in each cycle, where *P*_2i_ are 46400, 47200, 48000, 48800, and 49600, respectively. Additional unit cost for rework is $50, $55, $60, $65, and $70, respectively. During the rework process, there is a *failure in rework* rate of (*φ*_i_) 10%, 15%, 20%, 25%, 30% associated with each product. Units that fail during the rework process will be scrapped at a unit disposal cost of (*C*_Si_) $20, $25, $30, $35, and $40, respectively. Additional values for variables used in this example are listed as follows:

*C*_*i*_ = unit manufacturing costs are $80, $90, $100, $110, and $120 respectively.
*h*_*i*_ = unit holding costs are $10, $15, $20, $25, and $30 respectively.
*K*_*i*_ = production setup costs are $17000, $17500, $18000, $18500, and $19000, respectively.
*h*_1*i*_ = rework process unit holding costs are $30, $35, $40, $45, and $50, respectively.
*K*_1*i*_ = the fixed delivery costs per shipment are $1800, $1900, $2000, $2100, and $2200.
*h*_2*i*_ = unit holding costs at the sales offices are $70, $75, $80, $85, and $90 respectively.
*C*_T*i*_ = unit transportation costs are $0.1, $0.2, $0.3, $0.4, and $0.5 respectively.

Applying Eq (25), we obtain n* = 4.4122. In order to locate an integer value of n* (as discussed in Section 3, n* must be an integer), let *n*^+^ = 5 and *n*^*−*^ = 4, and plug them into Eq (24) one obtains (*T* = 0.6654, *n*^+^ = 5) and (*T* = 0.6183, *n*^*−*^ = 4). Applying Eq (16) with these two sets of policies one finds E[*TCU*(0.6654, 5)] = $2,280,154 and E[*TCU*(0.6183, 4)] = $2,279,874. To minimize the system cost, one selects (T* = 0.6183, n* = 4) as the optimal production-shipment policy for the proposed model and the long-run expected system cost is E[*TCU*(T*, n*)] = $2,279,874.

**Fig 4:**
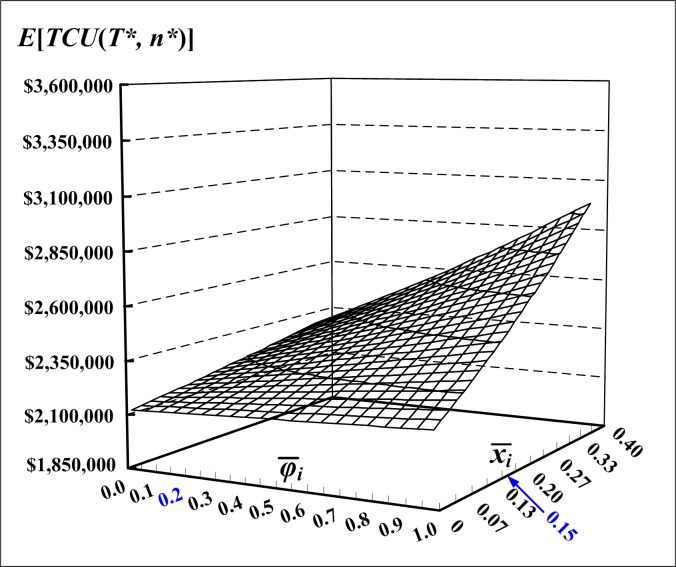
Variations of the mean failure-in-rework rate and mean defective rate and their effects on E[*TCU*(T*, n*)] of the proposed multi-product intra-supply chain system, which indicates how quality cost pushing the expected system cost higher

Fig 4 depicts variations of the mean failure-in-rework rate and mean defective rate and their effects on the expected system cost E[*TCU*(T*, n*)] of the proposed multi-product inventory system. It is noted that as the mean defective rate increases, E[*TCU*(T*, n*)] increases significantly, and as mean failure-in-rework rate increases, E[*TCU*(T*, n*)] increases slightly.

**Fig 5:**
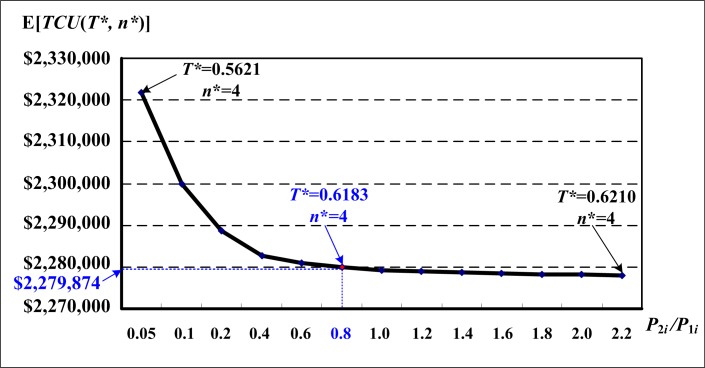
Variations of the ratio of rework and regular production rates (*P*_2i_/*P*_1i_) and their effects on the expected system cost E[*TCU*(T*, n*)], which shows that as *P*_2i_/*P*_1i_ decreases below a turning point 0.5, the expected system cost begins to increase significantly

The effect of the rework rate (in terms of the ratio of rework and regular production rates, i.e., *P*_2*i*_/*P*_1*i*_) on the expected system cost E[*TCU*(T*, n*)] are illustrated in Fig 5. It is noted that there is a turning point at ratio *P*_2*i*_/*P*_1*i*_ = 0.5 (i.e., when the time required to rework a nonconforming item is at least twice as long as the regular time needed to produce an item); as *P*_2*i*_/*P*_1*i*_ decreases below 0.5, the expected system cost E[*TCU*(T*, n*)] begins to increase significantly; and also from the turning point, as *P*_2*i*_/*P*_1*i*_ increases, E[*TCU*(T*, n*)] decreases slightly.

**Fig 6:**
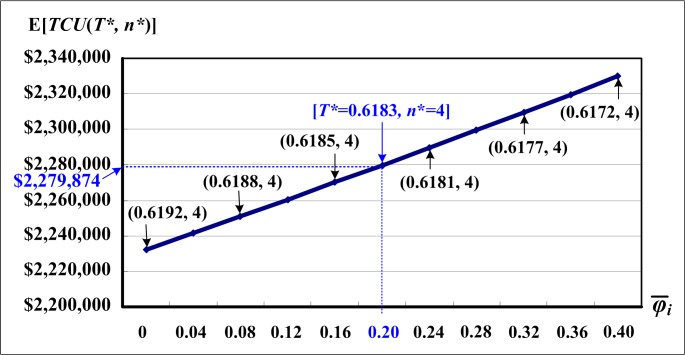
Variations of the mean failure-in-rework rates and their effects on optimal production-shipment policy and on the expected cost E[*TCU*(T*, n*)], which reveals that the proposed model is capable of deriving optimal solutions to given general real situations

Fig 6 illustrates the variations of mean failure-in-rework rates and their effects on the optimal production-shipment policy and on the expected cost E[*TCU*(T*, n*)]. It is noted that as mean failure-in-rework rate increases, the expected system cost E[*TCU*(T*, n*)] increases significantly, but the optimal rotation cycle time T* decreases slightly and n* is unchanged.

**Fig 7:**
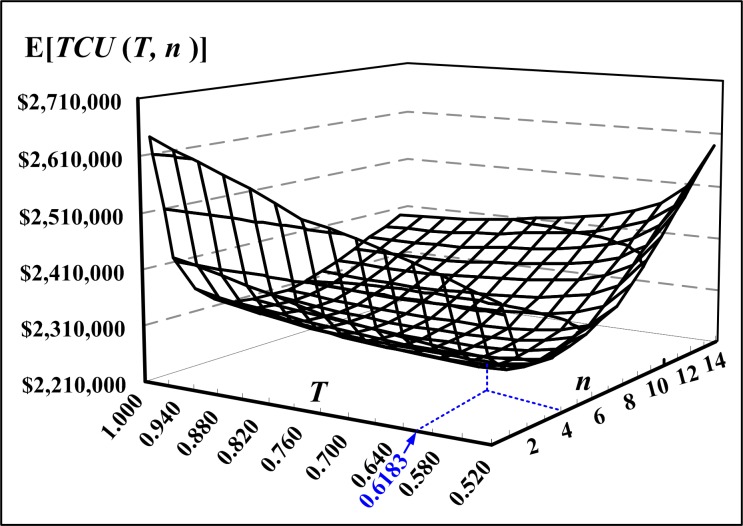
Variations of the common production cycle time *T* and number of deliveries *n* and their effects on E[*TCU*(*T*, *n*)], which illustrates the convexity of the expected system cost function

Fig 7 depicts the variations of the rotation cycle time *T* and number of deliveries *n* and their effects on the expected cost E[*TCU*(*T*, *n*)]. This example reconfirms the convexity of the expected cost E[*TCU*(*T*, *n*)].

**Fig 8:**
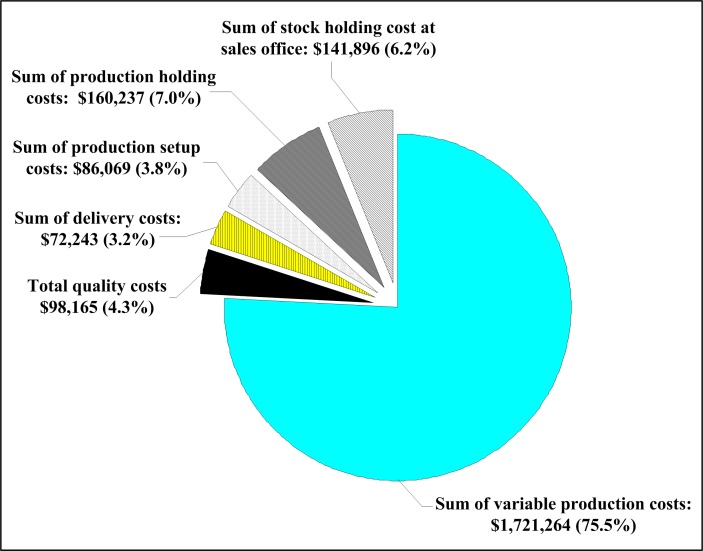
Detailed analysis of components of the expected system cost E[*TCU*(T*, n*)], which reveals the contributed percentages of each system related costs

Further analysis on the different components of the expected system cost E[*TCU*(T*, n*)] is displayed in Fig 8. It shows not only the dollar values of each cost components, but also their separate contributed percentages to the expected system cost. This can provide production managers with more insights of system cost parameters to assist them in cost control decision makings

## Conclusions

Optimization of a multi-product intra-supply chain system can benefit both production units and sales offices of an intra-supply chain’s parties, and help a firm achieve the goal of reducing operating cost. No wonder that it has recently drawn attention from management of the present-day transnational firms. This study developed an exact mathematical model to the multi-product intra-supply chain problem with failures in rework, with the objective is to jointly determine the common production cycle time and number of deliveries needed to minimize the expected production–inventory–delivery costs for the problem. Mathematical modeling and optimization techniques are used to help us derive the optimal decisions to the problem. The research results enable managers to achieve the operational goals of maximizing machine utilization, and reducing both quality and delivery costs of their intra-supply chain system. Through a numerical example, we demonstrate the applicability of research results and their practical improvements (see Figs 4–8) to the realistic intra-supply chain system. For future study, one may consider the effect of the variable production rates on the optimal common cycle length of the problem.

### Appendix A

Additional notations used in the proposed multi-product intra-supply chain system are as follows:

*Q*_i_ = production batch size per cycle for product *i*,
*t*_1i_ = uptime for product *i* in the proposed multi-product intra-supply chain system,
*t*_2i_ = rework time for product *i* in the proposed multi-product intra-supply chain system,
*t*_3i_ = delivery time for product *i* in the proposed multi-product intra-supply chain system,
*t*_ni_ = fixed interval of time between each installment of finished product *i* transported in *t*_3i_,
*T* = common production cycle time—a decision variable,
*H*_1i_ = maximum on-hand inventory level of product *i* when uptime ends,
*H*_2i_ = maximum on-hand inventory level of product *i* when rework process ends,
*d*_2i_ = production rate of scrap items during rework for product *i*,
*n* = number of fixed-quantity installments of the finished batch to be transported to sales office in each cycle—another decision variable,
*I*(*t*)_i_ = on-hand inventory level of perfect quality product *i* at time *t*,
*I*_c_(*t*)_i_ = on-hand inventory of product *i* stored at the sales offices’ at time *t*,
*D*_i_ = fixed quantity of finished product *i* transported to the sales offices per delivery,
*I*_i_ = left over product *i* per delivery after depletion during t_*ni*_,
*I*_s_(*t*)_i_ = on-hand inventory level of scrapped product *i* at time *t*,
*TC*(*Q*_i_,*n*) = total production-inventory-delivery cost per cycle for product *i*,
E[*TCU*(*Q*_i_,*n*)] = total expected production-inventory-delivery costs per unit time for producing *L* products in the proposed multi-product intra-supply chain system,
E[*TCU*(*T*,*n*)] = total expected production-inventory-delivery costs per unit time for producing *L* products in the proposed system using rotation cycle time as the decision variable.
